# Females have higher myocardial perfusion, blood volume and extracellular volume compared to males – an adenosine stress cardiovascular magnetic resonance study

**DOI:** 10.1038/s41598-020-67196-y

**Published:** 2020-06-25

**Authors:** Jannike Nickander, Raquel Themudo, Andreas Sigfridsson, Hui Xue, Peter Kellman, Martin Ugander

**Affiliations:** 1 Department of Clinical Physiology, Karolinska University Hospital, and Karolinska Institutet, Stockholm, Sweden; 2 Department of Radiology, Karolinska University Hospital, and Karolinska Institutet, Stockholm, Sweden; 3 National Heart, Lung, and Blood Institute, National Institutes of Health, Bethesda, MD USA; 4 Kolling Institute, Royal North Shore Hospital, and Charles Perkins Centre, Faculty of Medicine and Health, University of Sydney, Sydney, Australia

**Keywords:** Cardiovascular biology, Cardiovascular diseases, Acute coronary syndromes

## Abstract

Knowledge on sex differences in myocardial perfusion, blood volume (MBV), and extracellular volume (ECV) in healthy individuals is scarce and conflicting. Therefore, this was investigated quantitatively by cardiovascular magnetic resonance (CMR). Healthy volunteers (*n *= 41, 51% female) underwent CMR at 1.5 T. Quantitative MBV [%] and perfusion [ml/min/g] maps were acquired during adenosine stress and at rest following an intravenous contrast bolus (0.05 mmol/kg, gadobutrol). Native T1 maps were acquired before and during adenosine stress, and after contrast (0.2 mmol/kg) at rest and during adenosine stress, rendering rest and stress ECV maps. Compared to males, females had higher perfusion, ECV, and MBV at stress, and perfusion and ECV at rest (p < 0.01 for all). Multivariate linear regression revealed that sex and MBV were associated with perfusion (sex beta −0.31, p = 0.03; MBV beta −0.37, p = 0.01, model R^2^ = 0.29, p < 0.01) while sex and hematocrit were associated with ECV (sex beta −0.33, p = 0.03; hematocrit beta −0.48, p < 0.01, model R^2^ = 0.54, p < 0.001). Myocardial perfusion, MBV, and ECV are higher in female healthy volunteers compared to males. Sex is an independent contributor to perfusion and ECV, beyond other physiological factors that differ between the sexes. These findings provide mechanistic insight into sex differences in myocardial physiology.

## Introduction

In patients who present with acute coronary syndrome (ACS) women have, in general, a worse outcome when compared to men^1–3^. This discrepancy is multifactorial, and includes age differences, symptom presentation, and physiological differences. For example, women may present with symptoms of ischemic heart disease that differ compared to men, and thereby receive less aggressive treatment^4^, or have a higher vulnerability to psychological stress^5^, thus potentially explaining why women are more susceptible to myocardial ischemia secondary to psychological stress^5^. Coronary artery disease (CAD) and several non-ischemic heart diseases impair myocardial microcirculation^6^, and can be detected during adenosine stress first pass perfusion imaging using cardiovascular magnetic resonance (CMR)^7–11^. Recent developments in CMR allow fully automated acquisition and reconstruction of quantitative perfusion [ml/min/g], and myocardial blood volume (MBV) maps [%]^12^. The accuracy of the newly developed quantitative myocardial perfusion maps has been validated using the independent reference standard position emission tomography (PET) in clinical patients^13^. Furthermore, current knowledge on sex differences of normal myocardial physiology with regards to perfusion and MBV is scarce. However, any difference in normal range values for these physiologic parameters may impact the clinical identification of CAD in patients. Moreover, it is of importance to identify physiological sex differences in perfusion and MBV that may elucidate potential contributing factors to the sex differences in ACS and/or CAD. We therefore, sought to investigate if there are any sex differences in perfusion, MBV and extracellular volume (ECV) in normal physiology.

## Methods

### Study population

Healthy volunteers (*n* = 43, age mean ± SD 26 ± 5 years, 51% females) that were current non-smokers with a normal 12-lead ECG and no cardiovascular medication, history of asthma, cardiovascular or kidney disease were included. Exclusion criteria included failed splenic switch off^14^, low peak of contrast agent (<2 mmol/l), and poor image quality. This study cohort has previously been studied in order to evaluate the physiological contributions to magnetic relaxation properties of the myocardium unrelated to sex^15^.

### Image acquisition

Image acquisition was performed as previously described^15^, in brief CMR was performed with the volunteer supine at 1.5 T (Aera, Siemens Healthcare, Erlangen, Germany) using anterior and posterior phased-array surface coils. First, full coverage retrospective electrocardiographically gated balanced steady state free precession (bSSFP) cine imaging were acquired in short-axis, and three long-axis slices. Typical imaging parameters included flip angle (FA) 68°, pixel size 1.4 × 1.9 mm^2^, slice thickness 8.0 mm, repetition time (TR) = 37.05 ms, echo time (TE) = 1.19 ms, matrix size = 256 × 144 and field of view (FOV) 360 × 270 mm^2^.

Three short-axis slices were acquired using first pass perfusion imaging, both during adenosine stress (Adenosin, Life Medical AB, Stockholm, Sweden, 140 microg/kg/min infusion) and at rest, during administration of an intravenous bolus of contrast agent (0.05 mmol/kg, gadobutrol, Gadovist, Bayer AB, Solna, Sweden). Adenosine and the contrast agent were administered in separate cannulas. Perfusion and MBV maps were generated using the Gadgetron inline perfusion mapping software, and computed based on the distributed tissue exchange model (BTEX)^16^ as previously described^12^. Typical imaging parameters were: SSFP single shot readout, TE 1.04 ms, TR 2.5 ms, bandwidth 1085 Hz/pixel, FA 50°, FOV 360 × 270 mm^2^, slice thickness 8.0 mm, parallel acquisition technique factor 3 and saturation delay/trigger delay 95/40 ms.

**Figure 1:**
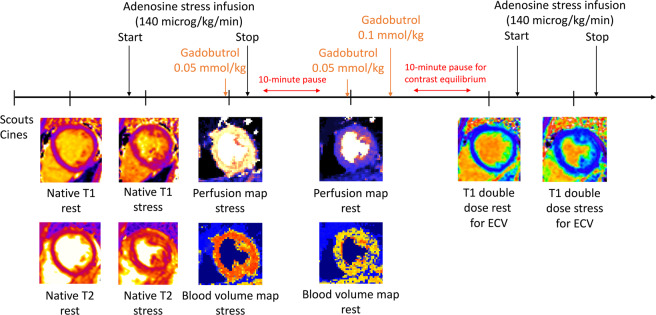
Timeline over CMR scan. Scouts and cines were acquired first, followed by native T1 and T2 mapping a rest. Following 3 minutes of adenosine infusion, native T1 and native T2 maps were acquired, followed by first pass perfusion imaging using gadobutrol (0.05 mmol/kg). After the adenosine infusion was terminated a 10-minute pause followed to reach contrast equilibrium. First pass perfusion images were acquired using gadobutrol (0.05 mmol/kg) at rest and an additional dose of gadobutrol (0.1 mmol/kg) was administered. Post-contrast T1 maps were acquired at rest, and at stress following 3 minutes of adenosine infusion. Reproduced from^15^ under the Creative Commons Attribution 4.0 International License (http://creativecommons.org/licenses/by/4.0/).

Three short-axis native T1 maps were acquired using an electrocardiographically-gated modified Look-Locker inversion recovery (MOLLI) sequence^17^ with a 5s(3s)3s sampling scheme^18^ (Siemens WIP 1041). The native T1 maps were acquired before and during adenosine stress (140 microg/kg/min infusion). Post-contrast T1 maps with the same slice position and sampling scheme as the native T1 maps, were acquired following intravenous contrast (total dose of 0.2 mmol/kg, consisting of 2 boluses each at 0.05 mmol/kg, followed by an additional 0.1 mmol/kg for ECV), both at rest and during adenosine stress (140 microg/kg/min infusion), see Fig. 1. The T1 maps were reconstructed using in-line motion correction^19^. Typical imaging parameters included SSFP single-shot readout in end-diastole, FA 35°, pixel size 1.4 × 1.9 mm^2^, slice thickness 8.0 mm, imaging duration 167 ms, TE 1.12 ms, matrix size = 256 × 144 and FOV 360 × 270 mm^2^. Rest and stress ECV maps was generated offline from rest pre- and post-contrast T1 maps, and stress pre and post-contrast T1 maps, respectively, and calibrated by blood hematocrit^20^.

### Image analysis

Perfusion, MBV and ECV maps were anonymized offline and analyzed with the freely available software (Segment software version 2.0 R5152, Medviso AB, Lund, Sweden) with the observer blinded to subject identity, sex, body size and age during image analysis. Perfusion, MBV and ECV average values were acquired by conservatively drawing a circumferential region of interest in the midmural third of the myocardium in the respective midventricular short-axis maps averaged over three slices, both at rest and stress.

Left ventricular (LV) volumes, ejection fraction and myocardial mass were quantified from the short-axis cine stack using the SyngoVia Software, VA30 (Siemens, Erlangen, Germany). Volumetric measurements and myocardial mass were indexed to body surface area according to the Dubois formula^21^. Myocardial mean left ventricular wall thickness, also known as global thickness (GT), was calculated using a validated formula which includes the input variables left ventricular mass and end-diastolic volume^22^, namely, GT = 0.05 + 1.60 * LVM^0.84^ * LVEDV^−0.49^.

### Statistical analysis

Quantification of myocardial perfusion, MBV, and ECV was performed in each slice, and global values per subject were acquired by averaging the values from all three slices in each subject. Continuous variables were reported as means together with their standard deviation (SD). Ordinal variables were reported as percentages. Mean values were compared by using the paired or unpaired t-test as appropriate in normally distributed data, as decided by the Kolmogorov-Smirnov test. The relationships between continuous variables were assessed with Pearson’s linear correlation coefficient, and presented as R^2^. Multivariable associations were evaluated using multivariate linear regression by entering a priori defined physiological covariables. Statistical analysis was performed using Microsoft Excel (Microsoft, Redmond, Washington, USA) and IBM SPSS Statistics (IBM SPSS Statistics 23, IBM, New York, USA). The significance level in all statistical analyses was defined as *p* < 0.05.

### Ethics, consent and permission

All study procedures were approved by the Regional Ethics Review Board in Stockholm, ID nr: 2015/2106-31/1 and all healthy volunteers provided written informed consent. All study procedures were carried out in accordance with relevant guidelines and regulations as per the Declaration of Helsinki for involving human participants. Clinicaltrials.gov identifier: NCT02723747, registered March 16, 2016.

### Consent to publish

Written informed consent was obtained from healthy volunteers for publication of their individual details on a group level and anonymized images in this manuscript.

## Results

### Study population

**Figure 2:**
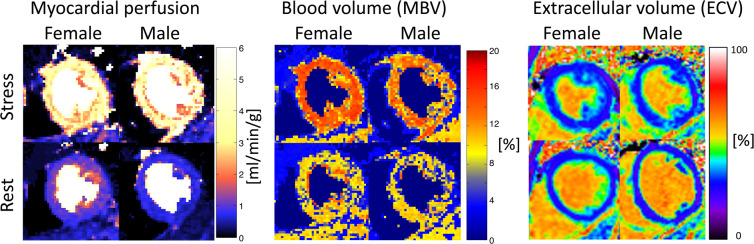
Examples of myocardial perfusion, myocardial blood volume and extracellular volume maps at rest and stress. The top panels show images acquired during adenosine stress and the bottom panel shows rest images. The left column shows a healthy female and the right a healthy male. Myocardial perfusion and myocardial blood volume (MBV) maps were acquired in systole, and extracellular volume (ECV) maps were acquired in late diastole. Note, the ECV maps may appear to be acquired in different slice positions, however this is due to the increase in heart rate during adenosine stress, which shortens diastole thus making the acquisition closer to systole, despite that the slices positions are the same in rest and stress. It is evident that the differences in myocardial perfusion, MBV and ECV are not visually apparent, however this further highlights the importance of proper quantification for correct diagnosis, which also underscores the important of correct normal reference values.

Two volunteers were excluded due to failed contrast injection (n = 1) and low contrast peak (n = 1), respectively. Table 1 shows baseline characteristics of the study cohort (*n* = 41). Stress ECV images could not be processed in 4 cases because pre and post T1 maps were mistakenly acquired with different FOV, and these cases were excluded from analysis of ECV. Figure 2 shows representative cases of a male and female with all respective maps.

**Table 1:** Baseline characteristics of healthy volunteers.

Characteristic	All (*n* = 41)	Females (*n* = 21)	Males (*n* = 20)
Female sex, *n* (%)	21 (51)		
Age, years	26 ± 5	25 ± 5	26 ± 6
Height, cm	175 ± 9	169 ± 7	181 ± 5*
Weight, kg	71 ± 11	65 ± 7	78 ± 9*
BSA, m^2^	1.9 ± 0.2	1.7 ± 0.1	2.0 ± 0.1*
LVEDV, ml	176 ± 36	154 ± 20	201 ± 35*
LVEDVI, ml/m^2^	94 ± 14	88 ± 10	102 ± 16*
LVESV, ml	72 ± 17	63 ± 11	82 ± 18*
LVESVI, ml/m^2^	39 ± 7	36 ± 5	41 ± 8*
LVSV, ml	104 ± 22	91 ± 14	119 ± 20*
LVSVI, ml/m^2^	56 ± 9	52 ± 8	60 ± 9*
LVEF, %	59 ± 4	59 ± 4	59 ± 4
LVM, g	119 ± 28	99 ± 13	140 ± 22*
LVMI, g/m^2^	63 ± 11	57 ± 7	70 ± 9*
GT, mm	7.0 ± 0.8	6.5 ± 0.5	7.6 ± 0.7*
Rest ECV, %	27 ± 3	29 ± 2	25 ± 3*
Hematocrit, %	42 ± 4	39 ± 3	45 ± 3*

Data presented as mean ± SD. Abbreviations: EDV – end diastolic volume; ESV – end systolic volume; SV – stroke volume; EF – ejection fraction, LVM – left ventricular mass. GT – global thickness * denotes p < 0.05 for differences between the sexes.

### Myocardial perfusion

**Figure 3:**
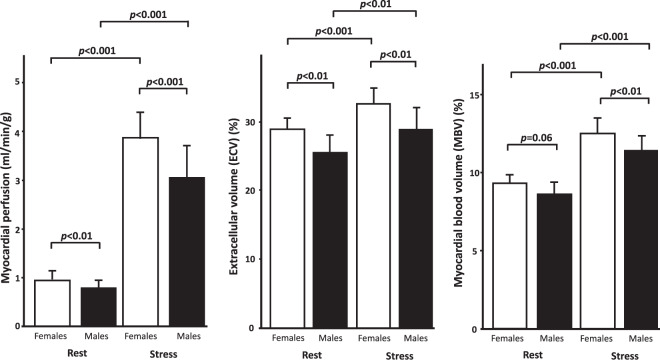
Sex differences in myocardial perfusion, extracellular volume and myocardial blood volume at rest and stress. Females had higher perfusion at both rest and adenosine stress compared to males. Females had higher extracellular volume at both rest and adenosine stress compared to males. Females had higher myocardial blood volume at adenosine stress compared to males, however not at rest. Error bars show one standard deviation. *p-*values denote paired or unpaired t-test as appropriate.

Myocardial perfusion at rest for all subjects was 0.88 ± 0.19 ml/min/g and at stress 3.62 ± 0.71 ml/min/g (*p* < 0.001 compared to rest). Females had higher perfusion both at rest and stress compared to males (rest 0.95 ± 0.18 vs 0.80 ± 0.17, *p* < 0.01; stress 4.0 ± 0.57 vs 3.22 ± 0.64 ml/min/g, *p* < 0.001), Fig. 3.

### Extracellular volume

Mean ECV for all subjects at rest was 27 ± 3 and at stress 31 ± 3% (*p* < 0.001). Females had higher ECV both at rest and stress compared to males (rest 29 ± 2 vs 25 ± 3, *p* < 0.01; stress 33 ± 2 vs 29 ± 3%, *p* < 0.01), Fig. 3.

### Myocardial blood volume and extracellular volume fraction

**Figure 4:**
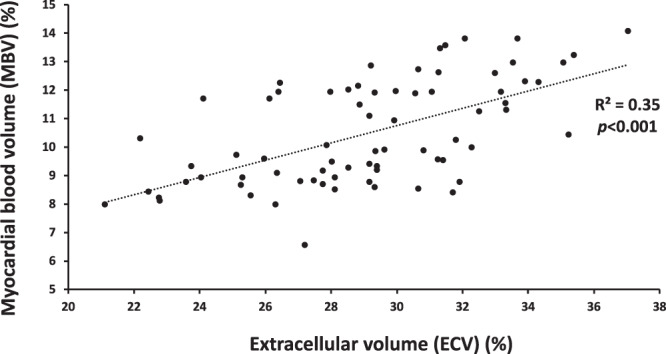
Linear correlation between myocardial blood volume and extracellular volume. The equation describing the linear correlation between extracellular volume (ECV) and myocardial blood volume (MBV) was ECV = 1.1 × MBV + 17.

**Figure 5:**
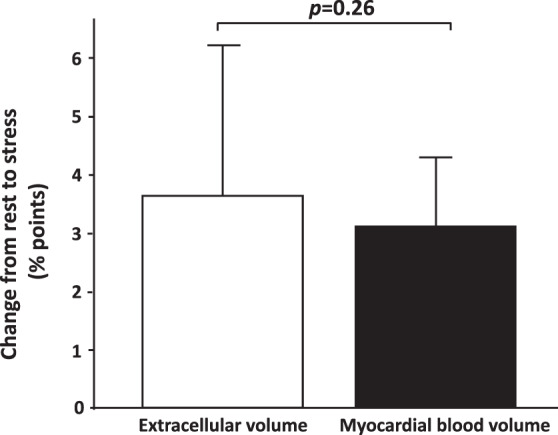
Stress-rest increase of myocardial blood volume and extracellular volume. There was no difference in stress-rest increase of myocardial blood volume and stress-rest increase of extracellular volume in all healthy volunteers, *n* = 37. Error bars show one standard deviation. *p-*value denote paired t-test.

MBV for all subjects at rest was 8.6 ± 0.6 and at stress 11.8 ± 1.1% (*p* < 0.001). Females had higher MBV at stress compared to males (12.6 ± 0.9 vs 11.5 ± 0.9%, *p* < 0.01), however not at rest (8.8 ± 0.5 vs 8.4 ± 0.7, p = 0.06), Fig. 3. MBV and ECV were correlated (R^2^ = 0.35, *p* < 0.001), Fig. 4. There was no difference in the mean rest-to-stress increase in MBV compared to the mean rest-to-stress increase in ECV (3.7 ± 2.6 vs 3.1 ± 1.2 percentage points, *p* = 0.26), Fig. 5.

### Rate pressure product and coronary flow reserve

There was no difference in heart rate, blood pressure, rate pressure product (RPP) or myocardial perfusion reserve (MPR) between the sexes, see Table 2. Since there was no difference between the sexes, RPP was not investigated further.

**Table 2:** Rate pressure product and myocardial perfusion reserve across the sexes.

Characteristic	All (*n* = 41)	Females (*n* = 21)	Males (*n* = 20)	p-value
Rest HR, bpm	71 ± 12	71 ± 10	71 ± 15	0.86
Stress HR, bpm	95 ± 16	98 ± 15	91 ± 18	0.23
Rest RPP, bpm*mmHg	7815 ± 1893	7533 ± 1453	8115 ± 2266	0.34
Stress RPP, bpm*mmHg	10609 ± 2451	10638 ± 2266	10579 ± 2691	0.94
Change in RPP, %	38 ± 28	43 ± 30	33 ± 25	0.25
MPR	4.2 ± 1.0	4.3 ± 1.0	4.2 ± 1.1	0.64

Data presented as mean ± SD. Abbreviations: MPR – myocardial perfusion reserve; HR- heart rate; RPP – rate pressure product.

### Explanatory factors

In order to understand the sex differences observed in MBV, ECV and perfusion, parameters that differed between the sexes were investigated using multivariate linear regression. These evaluated parameters that differed according to sex were: height, weight, BSA, LVEDVI, LVESVI, LVSVI, LVMI and GT. In multivariate analysis, none of these parameters were both associated with either MBV, perfusion, or ECV at rest and stress, respectively (data not shown). Consequently, the physiological parameters MBV, myocardial perfusion, ECV and hematocrit were selected to be assessed separately together with sex at rest and stress, respectively, and the results are summarized in Table 3. Sex and MBV were independent contributors to perfusion at both rest and stress. Sex and hematocrit were independent contributors to ECV at rest. The only contributor to MBV was perfusion at both rest and stress.

**Table 3:** Multivariate linear regression analysis.

Perfusion	Rest Global R^2^ = 0.29, p < 0.01		Stress Global R^2^ = 0.42, p < 0.001	
	ß	p-value	ß	p-value
Sex	−0.31	0.03	−0.43	0.01
ECV	0.10	0.60	0.34	0.83
MBV	0.37	0.01	0.37	0.01
Hematocrit	0.18	0.44	0.06	0.77
**MBV**	**Rest Global R**^**2**^** = 0.20, p < 0.01**		**Stress Global R**^**2**^** = 0.26, p < 0.01**	
Sex	−0.06	0.81	0.04	0.84
ECV	−0.23	0.30	−0.03	0.90
Perfusion	0.45	0.01	0.50	0.01
Hematocrit	−0.20	0.20	−0.12	0.45
**ECV**	**Rest Global R**^**2**^** = 0.54, p < 0.001**		**Stress Global R**^**2**^** = 0.42, p < 0.001**	
Sex	−0.33	0.03	−0.25	0.13
Perfusion	0.12	0.41	0.04	0.77
MBV	−0.10	0.41	−0.02	0.90
Hematocrit	−0.48	0.01	−0.65	0.01

Abbreviations: MBV – myocardial blood volume.

## Discussion

This study demonstrates that myocardial perfusion, MBV, and ECV are all higher in females compared to males during adenosine stress CMR, and that sex is an independent contributing factor to myocardial perfusion and ECV. These results provide insights into sex differences in these physiological characteristics of the myocardium. Furthermore, this illustrates the importance of sex-specific normal reference values for perfusion, MBV and ECV, which may contribute to a higher accuracy of CMR in the diagnosis and follow-up of patients with CAD. A recent study suggested that hyperemic myocardial perfusion [ml/min/g] is non-inferior to other methods for determining functionally relevant coronary stenosis^23^, and following that women have higher perfusion, a sex-specific normal reference value may increase accuracy in CAD diagnosis by CMR. As there is no difference in MPR, sex-specific normal values would only be needed for absolute rest and stress perfusion values. Furthermore, these findings might contribute to a better understanding of the sex differences in cardiovascular mortality and morbidity between males and females. Sex differences in perfusion have previously been shown in positron emission tomography (PET) studies in a variety of populations (i.e. diabetics, heart failure)^24–29^, thus this study supports that females have higher perfusion compared to males, both at rest and at stress. Importantly, this is the first study to definitively show that sex is an independent contributor to both myocardial perfusion and ECV, beyond parameters such as BSA, LV volumes, and hematocrit.

The current non-invasive reference standard for quantifying absolute perfusion is PET^30,31^. However, PET has some drawbacks including radiation exposure and limited spatial resolution. CMR first pass perfusion imaging has excellent agreement with both PET^11^ and microspheres^32^. Furthermore, global perfusion can also be quantified through coronary sinus flow by CMR^33,34^. In one study^34^, absolute rest perfusion was 1.0 ± 0.1 ml/min/g, as quantified by coronary sinus flow CMR imaging, which corresponds closely to the quantified rest perfusion in the current study of 0.9 ± 0.2 ml/min/g. Like PET images, quantitative perfusion maps are computed based on some sort of tissue exchange model or compartment model. Quantitative perfusion CMR in this study used a BTEX model^16^, implemented in the Gadgetron framework, that estimates a total of 5 parameters. The software employing this BTEX model is freely available as an executable^35^, and it estimates arterial delay, perfusion, MBV, permeability surface area and interstitial volume. The perfusion acquisitions are set to 60 measurements, limiting the precision of the estimated interstitial volume, which indeed is not the purpose of the perfusion scan, however the interstitial volume parameter from this BTEX model of the perfusion analysis has previously been shown to be in reasonable agreement with T1-based estimates of ECV^35^. Furthermore, there is a very low correlation between the interstitial volume and perfusion from the quantitative perfusion scan (data not shown). Simpler models, such as the Fermi model^36^ generally estimates 4 parameters, including arterial delay. Adding an additional parameter has not significantly changed the stability of the estimates, and has the added benefit of properly handling the extraction fraction^35^. In a study in healthy volunteers^29^, females had higher rest myocardial perfusion compared to males, which supports the main finding of sex differences in our study. Sex differences in myocardial perfusion have previously been reported in several patient populations^24–28^. In a sizeable cohort of patients without known CAD and a normal stress PET scan (n = 1218), women had higher myocardial perfusion compared to men^37^. It has been suggested that sex differences are largely due to that women in general have thinner myocardium, and through partial volume effects there is blood pool contamination that accounts for the higher values in women, as both PET and single positron emission tomography has limited spatial resolution. CMR has better spatial resolution, and furthermore, sex difference in T1 values are not primarily due to myocardial wall thickness, but varies with hematocrit^38^, thus contradicting the notion that sex differences are only due to differences in myocardial wall thickness. However, in the study by Range *et al*. (27), there was no difference in perfusion between males and females at adenosine stress, which is in contrast to the findings in the current study. In that study, the volunteers were older (mean age 34 years) compared to the healthy volunteers in the current study (mean age 26 years). As the sex differences in cardiovascular mortality and morbidity decrease with age^39^, the age difference could contribute to this discrepancy.

In an attempt to understand the observed sex differences in this study we investigated other parameters that differed between the sexes, such as BSA and LV volumes, with regards to their impact on MBV, perfusion and ECV. None of the parameters contributed to MBV, perfusion or ECV both during rest and during stress, respectively. Consequently, we continued to investigate the physiological parameters and sex for their contributions at rest and stress. Sex was a multivariate independent contributor to both ECV and perfusion at rest, and to perfusion at stress. Hematocrit contributed to ECV at both rest and stress. The only contributor to MBV was perfusion at both rest and stress. Importantly, these findings suggest that sex is an important physiological parameter in myocardial ECV and perfusion, and that the sex differences observed in this study are not due to differences in body size, LV volumes or hematocrit alone. The sex differences observed in this study of normal physiology raise several mechanistic questions regarding the healthy female myocardium. Given that females have higher ECV it has been suggested that females have greater space between the cardiomyocytes, which could be an explanation for the need for higher resting perfusion. However, higher ECV and higher myocardial perfusion could also be a result of higher capillary density in females, which would provide a unifying physiological explanation. A study of myocardial capillary density in healthy myocardium across different ages is currently being undertaken to try to address this question.

MBV can be accurately measured by first pass perfusion imaging CMR^40^, and myocardial contrast echocardiography can quantify both perfusion^41,42^ and MBV^43^. However, the relationship between myocardial vasculature, blood flow and blow volume has not been completely elucidated^40,44,45^, and cumbersome methods for *in vivo* quantification of these variables in humans complicates the validation of MBV imaging methods^46^. ECV is expected to increase during adenosine stress if there is an increase in myocardial blood volume, due to the resulting increase in extracellular space that occurs when the myocardial plasma volume increases concomitantly with the increased MBV. The change in ECV values between rest and stress are therefore partially caused by the increase in MBV between rest and stress. There was no difference between the rest-to-stress increase in MBV and the rest-to-stress increase in ECV, thus supporting the notion that the stress-induced changes in the MBV maps indeed reflect stress-induced changes in ECV. Changes in ECV provide independent validation of the accuracy of stress-induced changes in MBV. The physiological parameters ECV, MBV, and myocardial perfusion have been shown to be closely interlinked, and their intrinsic relationships have not been completely elucidated^40,44,45^. However, multivariate linear regression is, by definition, the statistical method used to determine whether or not two variables such as ECV and perfusion have independent associations with sex, respectively. Since assessment of collinearity is performed as a part of the multivariate analysis, it is possible to conclude that sex indeed is an independent contributor to myocardial perfusion and ECV. Furthermore, ECV was quantified with an independent method compared to myocardial perfusion and MBV, adding additional support to the conclusion that sex differences in normal myocardial physiology exist.

This study was conducted in a small cohort of young healthy volunteers without any cardiovascular disease. As the study population was young, we donot know if sex differences exist in other age groups. Furthermore, sex was the primary characteristic investigated. Other characteristics of the study population could potentially contribute to the observed sex differences, including level of physical activity or ethnicity, which previously has been discussed^15^ but were not expressly assessed in the current study. Also, only global values were investigated (per-subject averaging over 3 slices), which may be an over simplification of the physiology across the entire LV. However, the fact that sex differences persist despite averaging supports that there are sex differences across all the physiological parameters that exist on a global level. Only the mid-mural third of the myocardium was investigated, which may be an oversimplification as recent studies suggest transmural heterogeneity of myocardial perfusion^47,48^. However, drawing upon previously work in native T1 maps, there were no sex differences in native T1 due to myocardial wall thickness when examining the mid-mural third of myocardium^38^, instead the observed sex differences in native T1 were only due to differences in hematocrit. As the voxel size is similar between perfusion and native T1 maps, and the sex differences exist both at rest and stress, the current results suggest that perfusion in the mid-mural third of the myocardium does not differ between the sexes due to myocardial wall thickness. Importantly, there was no correlation between left ventricular global thickness (GT) and perfusion or MBV at either rest or stress. GT was associated with ECV both at rest and stress, respectively. However, this association did not persist in multivariate analysis when sex was included in the model. Regardless, sex differences were found when using the described methodology with analysis performed blinded to sex, and therefore the analysis methodology as such is unlikely to have contributed to the sex differences. The use of ECV mapping as a validation method for the stress-induced changes could potentially have introduced some measurement errors, due to incorporation of two separately acquired slices (pre- and post-contrast T1 maps, respectively) into one measurement. Furthermore, post-contrast T1 maps were acquired with a 5s(3s)3s MOLLI scheme, due to clinical routine at the time of this study, and not 4s(1s)3s(1s)2s which is currently recommended for shorter T1 values^18^, which theoretically may lead to a very small overestimation of ECV.

In conclusion, myocardial perfusion, MBV, and ECV are higher in female healthy volunteers compared to males. ECV measured at rest and stress provide independent validation of the accuracy of stress-induced changes in MBV. Sex is an independent contributor to myocardial perfusion and ECV, beyond other physiological factors that differed between the sexes. These findings provide mechanistic insight into sex differences in myocardial physiology.
